# CEP55 Inhibitor: Extensive Computational Approach Defining a New Target of Cell Cycle Machinery Agent

**DOI:** 10.34172/apb.2022.021

**Published:** 2021-01-31

**Authors:** Beni Lestari, Rohmad Yudi Utomo

**Affiliations:** ^1^Cancer Chemoprevention Research Center, Faculty of Pharmacy, Universitas Gadjah Mada, Sekip Utara, Yogyakarta, 55281, Indonesia.; ^2^Laboratory of Medicinal Chemistry, Department of Pharmaceutical Chemistry, Faculty of Pharmacy, Universitas Gadjah Mada, Sekip Utara, Yogyakarta, 55281, Indonesia.

**Keywords:** Anticancer, Evolutionary analysis, Molecular docking, CEP55 inhibitor, Natural product

## Abstract

***Purpose:*** Centrosomal protein 55 (CEP55) is a pivotal protein for cytokinesis during cell division. This study aimed to provide a comprehensive information about the CEP55 gene, including its expression pattern in several cancer types, conduct functional domain analysis across species, and perform a computational approach for potential inhibitors of CEP55.

***Methods:*** The expression levels of CEP55 in different cancers were analyzed using the Oncomine and TCGA databases. Evolutionary analysis of the CEP55 gene in various species was performed using MEGA-X software. Molecular docking analysis was used to screen the binding affinity of several natural products on CEP55–ALIX binding interaction.

***Results:*** High CEP55 expression was observed in 16 datasets of different cancer types. The high expression of the CEP55 protein was associated with worse outcomes in cancer treatments. Phylogenetic and evolutionary analyses revealed that the amino acid residues essential for CEP55 binding and localization were mostly conserved across vertebrates. Seventeen plant-based compounds were docked against the CEP55 protein to determine their binding affinities and illustrated specific sites of interaction for predicting novel protein–drug interactions. Flavanol compounds epigallocatechin gallate and catechin possessed superior binding affinity to all other compounds owing to the substitution of gallic ester or hydroxyl groups on the C3 position.

***Conclusion:*** This study provides comprehensive information about the CEP55 gene and insights for designing potent inhibitors against CEP55 signaling.

## Introduction

Among many anticancer drugs, drugs targeting mitosis are widely used in clinical application.^1^ However, anti-mitotic drugs possess several side effects, mainly peripheral neuropathy, because they also attack non-dividing cells.^2^ Therefore, drugs targeting other non-structural but essential mitotic machineries are urgently needed to treat cancer. Mitosis is the shortest phase of the cell cycle, but it is responsible for cytokinesis or the division of the replicated nuclear material in parallel with the physical cleavage of two daughter cells.^3^ Cytokinetic abscission, which occurs in the midbody, requires a series of proteins for successful separation of two daughter cells.^4^

Centrosomal protein 55 (CEP55) or centrosomal protein of 55 kDa is the key player of membrane abscission during cytokinesis. CEP55 is a coiled-coil protein (2–7 alpha-helices are coiled together) that contains 464 amino acids and forms a homodimer through the two-coiled region (CC1 and CC2).^5,6^ Accordingly, CEP55 has different subcellular localizations depending on the function, from the centrosomes during interphase to the midbody, the site of membrane abscission during mitosis. In the midbody, CEP55 recruits endosomal sorting complex required for transport machinery (ESCRT) components by direct interaction with ESCRT-I subunit members TSG101 (tumor susceptibility gene 101) and ALIX (ALG-2-interacting protein X) through the EABR domain, then leads to the recruitment of ESCRT-III for completing cytokinesis.^7,8^ Overexpression has been found in various tumors related to poor prognosis in cancer patients. The upregulation of CEP55 promotes genomic instability and tumorigenesis in mice. *In vitro* studies found that CEP55 overexpression induces uncontrolled cell proliferation via the PI3K/Akt signaling pathway.^9^ By contrast, CEP55 deregulation is associated with incomplete and abnormal cell division.^10,11^ The desired outcome of this anticancer strategy is that the inhibition of CEP55 activity results in aberrant cell division followed by cancer cell death. Therefore, this study provides comprehensive information about the CEP55 gene, including its expression, functional domains, important phosphorylation sites, and unfavorable clinicopathological features of abnormal expression.

Research for anticancer drugs has proven that natural products are a leading source of new anticancer synthetic compounds. Over 60% of anticancer drugs approved by the FDA are plant-derived natural products, such as vinca alkaloids (vincristine and vinblastine) and taxanes (paclitaxel), known as anti-mitotic drugs.^12,13^ Natural products play an important role in new drug discoveries and new drug-lead compounds because of their unique active ingredients.^14,15^ In the present study, we confirmed the characteristic interaction between CEP55 and ALIX, which is essential for CEP55 function. We also attempted to screen several plant-based compounds entering clinical trials against the CEP55 protein to look for potential CEP55 inhibitors.

## Materials and Methods

### Dataset collection

The following seventeen plant-based compounds entering clinical trials were obtained from the literature and clinical trial database: catechin, colchicine, curcumin, daidzein, epigallocatechin gallate (EGCG), genistein, hesperetin, hesperidin, homoharringtonine, ingenol mebutate, luteolin, naringenin, paclitaxel, quercetin, resveratrol, vinblastine, and vincristine.^16^ The chemical structures were drawn using ChemDraw software and then subjected to conformational search and energy minimization in MOE.

### Oncomine analysis

An online public database of microarray profiles and next-generation sequencing, Oncomine, was used (https://www.oncomine.org) to analyze the CEP55 gene expression in several types of cancers. The comparison of expression levels was performed by carrying out cancer vs. normal analysis. Subsequently, box plots were recreated using MS Excel. All statistical values were obtained directly from the corresponding database. The threshold for statistical significance was set as *P* < 0.01; fold change > 2; and gene rank, top 10%.

### The Cancer Genome Atlas analysis

Genetic events of CEP55 in many cancer types were analyzed using The Cancer Genome Atlas (TCGA) database. The TCGA CEP55 data set was retrieved from the TCGA database using the cBioPortal website (http://www.cbioportal.org). The analysis was shown as a graph of alteration frequency including “Amplification” and “Deep Deletion” in several cancer types.

### Phylogenetic analysis and amino acid sequence alignment

Evolutionary analysis of CEP55 including amino acid sequence alignment and phylogenetic tree in various mammalian and non-mammalian vertebrates was performed using MEGA-X (Molecular Evolutionary Genetic Analysis) software. The amino acid alignment was performed using ClustalW, and a phylogenetic tree analysis was constructed using the neighbor joining method. Reference sequences of various species were obtained from the National Center for Biotechnology Information (https://www.ncbi.nlm.nih.gov) and ENSEMBL (https://www.ensembl.org) databases.

### Molecular docking

Molecular docking analysis was performed to screen the binding affinities of several natural products on CEP55–ALIX binding interaction. Molecular docking simulation, RMSD calculation, and visualization of binding interaction were conducted using MOE 2010.12 (Licensed from Faculty of Pharmacy UGM).^16,17^ The PDB ID 3E1R was used as the model of CEP55 considering the available complex with ALIX. The molecular docking screening system used a flexible ligand and a rigid receptor. The site finder mode in MOE was used to determine the possible binding site for molecular docking screening. The structure of all compounds was drawn in ChemDraw software by inputting the smiles code in PubChem (https://pubchem.ncbi.nlm.nih.gov), and then the conformation series were generated in MOE. The default settings in MOE for molecular docking simulation were used unless further modification was available. The lowest docking score for each compound was collected as the possible binding interaction on CEP55.

## Results and Discussion

### Structure and molecular functions of CEP55

**Figure 1:**
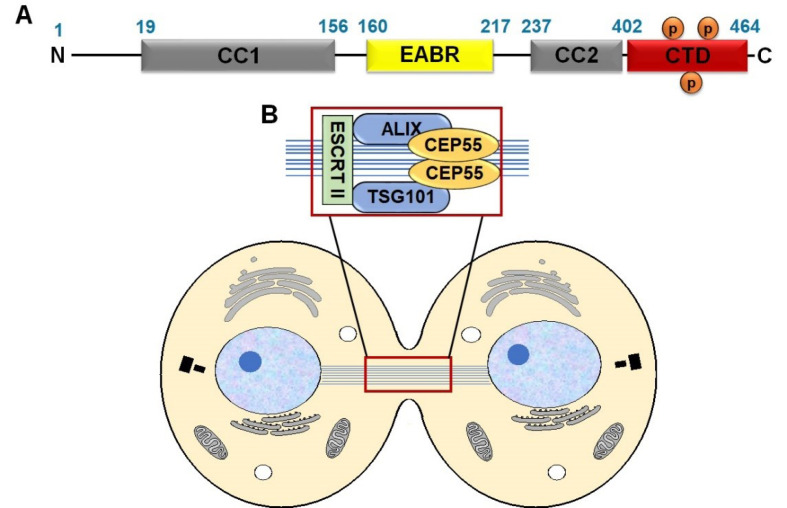
Schematic illustration of the CEP55 domains (A) and the assembly of CEP55-ALIX and ESCRT machinery during late cytokinesis (B). CC1: coiled-coil structure; CTD: C-terminal domain; EABR: ALIX-binding region; ESCRT: Endosomal sorting complex required for transport; ALIX: ALG-2-interacting protein X and TSG101: tumor susceptibility gene 101, components of ESCRT I complex; TEX14: Testis-expressed gene 14.

CEP55 is composed of well-defined domains, including coiled-coil structure, CC1 (19–156 bp) and CC2 (237–402 bp), which play a key role in dimerization. The EABR domain is located in the middle region as an ALIX interaction site (160–217 bp). The C-terminal domain (CTD: 402–464 bp) mediates CEP55 localization to midbody and contains several phosphorylation sites, especially Ser425, Ser428, and Ser436^18^ (Figure 1A). CEP55 localizes at the centrosome during interphase and moves to the midbody during anaphase. The change in CEP55 localization is mediated by the phosphorylation of Ser425 and Ser428 in the C-terminus by Cyclin-dependent kinase 1 (CDK1) and/or extracellular signal-regulated kinase 2 (ERK2), subsequent another phosphorylation at Ser436 by the polo-like kinase 1 (PLK1) protein.^19^ The second phosphorylation requires the first two phosphorylations and is responsible for the function of CEP55 in cytokinesis.^19,20^ In the midbody, CEP55 recruits ESCRT components by direct interaction with ESCRT-I TSG101 and ALIX through the EABR domain, subsequently induce ESCRT-III assembly.^7^ CEP55, ALIX, TSG101, and ESCRT III form protein complexes and decide whether to cut or uncut the dividing cells^21^ (Figure 1B).

### Expression of CEP55 in several types of cancer

**Figure 2:**
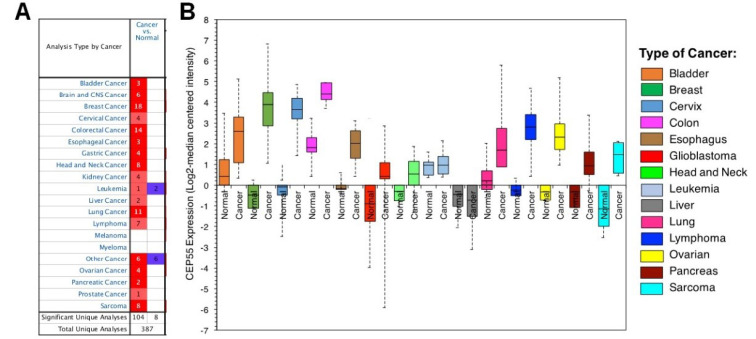
CEP55 gene expression analysis across different cancers relative to the normal tissue as revealed by Oncomine database. A. Box-whisker plots represent the differences in transcript levels of CEP55, between normal and tumor samples. The median value is represented by the middle line of the boxes. B. Expression pattern analysis of CEP55 mRNA in different cancer between tumor and normal tissues. The number of datasets with statistically significant (*P* < 0.01) CEP55 mRNA are indicated in the colored cells; overexpression (red) or reduced expression (blue). The gene rank was depicted by the color depth in the cells. The thresholds selected for inclusion of studies from the database were as follows: *P* value ≤ 0.01, fold change ≥ 2-fold and gene rank ≤ 10%.

**Figure 3:**
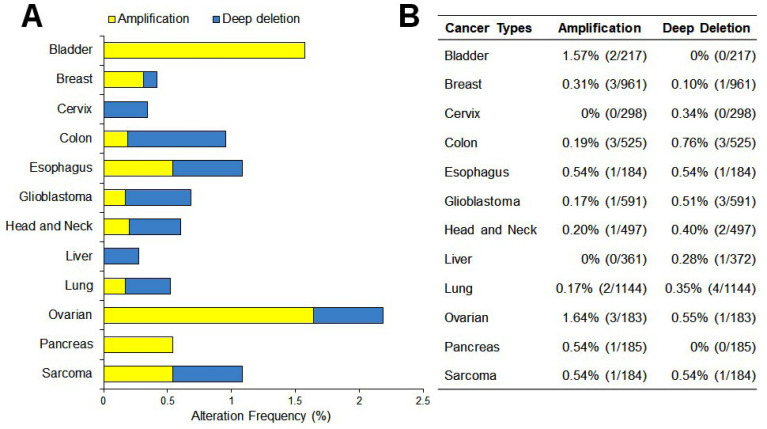
Genetic events of CEP55 in many cancer types. A. Profile of gene amplifications and gene deletions as determined by genomic profiling in The Cancer Genome Atlas (TCGA). B. The percentages shown reflect the overall rate of gene amplification and deletion in each cancer type as the number of the cases.

Studies from the Oncomine datasets presented a higher expression of CEP55 in a wide range of tumors compared with normal tissues. Figure 2A shows 387 unique analyses for CEP55 overexpression found in 16 datasets of different cancer types, whereas downregulation is observed only in two sets. Box plot analysis shows the upregulation of CEP55 in various human malignancies, including bladder cancer, breast cancer, cervical cancer, colon cancer, esophagus, glioblastoma, head and neck cancer, lung cancer, lymphoma, ovarian cancer, pancreatic cancer, and sarcoma, but not in leukemia and liver (Figure 2B). We further analyzed the CEP55 mRNA levels in the TCGA database via cBioPortal to confirm the results of Oncomine analysis. As shown in Figure 3, the TCGA database summarizes that the “amplification” of the CEP55 gene more frequently occurs than the “deletion” events in almost all cancer types. These findings are in agreement with the Oncomine dataset analysis, showing a high expression of CEP55 in many cancer types.

### Correlation between upregulation of CEP55 and clinical features of cancer patients

Previous studies of aberrant phenotypes and prognostic significance in the altered expression of CEP55 are summarized in Table 1. CEP55 overexpression correlates with the cancer aggressiveness and poor prognosis of patients with breast cancer.^6,22,23^ In cervical cancer, CEP55 protein levels are elevated in tumors as compared with adjacent normal tissue and correlated with lymph node metastasis and advanced tumor stage.^24^ Another study on adrenocortical carcinoma found that CEP55 is expressed at high levels and contributes to therapeutic failure.^25^ The copy number aberration of CEP55 has also been reported in bladder cancer,^26^ head and neck cancer,^27^ lung cancer,^28^ and pancreatic cancer.^29^ Those studies may be attributed to the fact that CEP55 is involved in cancer progression and correlated with unfavorable clinical features, highlighting its potential as a promising therapeutic target and predictive biomarker.

**Table 1:** CEP55 upregulation correlates with unfavorable clinicopathological parameters and poor prognosis in cancer

**Cancer Type**	**Detection Method**	**Clinicopathological Parameters or Phenotypes**	**Reference**
Adrenocortical carcinoma	Microarray, PCR, WB, IHC	CEP55 overexpression correlates with low comprehensive survival, disease-free survival, and pathology stage	25
Breast cancer	WB, Microarray	CEP55 overexpression promotes the survival of aneuploid cells and mediates resistance to docetaxel-induced apoptosis	6
	WB, RT-qPCR, Microarray	CEP55 knockdown inhibits cell proliferation and colony formation	22
WB, RT-qPCR, Microarray	CEP55 overexpression associates with aggressiveness in triple-negative breast cancer	23
Bladder cancer	RT-qPCR, IHC	CEP55 expression is higher in muscle-invasive bladder cancer than in non-muscle-invasive bladder cancer and is specific to transitional cell carcinoma of human urinary bladder	26
Cervical cancer	RT-qPCR	CEP55 overexpression associates with lymph node metastasis and advanced tumor stage	24
Head and Neck	RT-qPCR, IHC, Microarray	CEP55 shares a progressive expression pattern during the progression of head and neck squamous carcinoma	27
Lung cancer	Microarray	CEP55 overexpression correlates with the prognosis of lung adenocarcinoma	28
Pancreatic cancer	WB, RT-qPCR, IHC	CEP55 overexpression promotes PANC cells proliferation, migration, and invasion in vitro	29

### Understanding CEP55–ALIX binding for SiteFinder screening

**Figure 4:**
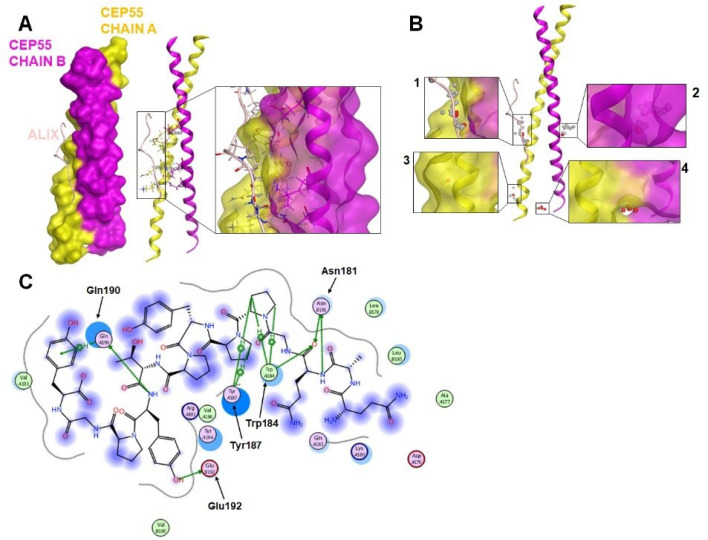
Interaction of CEP55 and ALIX through EABR domain. A. The overall structure of ALIX (pink, line ribbon) bound to CEP55 (Chain A: yellow, coil ribbon; Chain B: purple, coil ribbon). B. The location of druggable sequence generated by SiteFinder. C. Ligand Interaction view of CEP55-ALIX binding.

Recently, the discovery for the inhibitor of protein–protein interaction has shifted to the utilization of plant-derived natural products. The crystallography study of CEP55 highlighted the importance of ALIX on cytokinesis induction and then was inversely regulated by TEX14 (Testis-expressed gene 14). Therefore, our deduction attempted to identify a possible way of disrupting CEP55–ALIX interaction. A recent study has reported that ALIX interacts with CEP55 homodimer via its EABR domain (Figure 4A). In particular, our protein interaction analysis using MOE showed the involvement of three H-bond and five arene-H bonds, indicating the domination of hydrophobic bonding on CEP55–ALIX interaction (Table 2). Three Arene-H bonds formed between Tyr187 from CEP55 chain A and Pro801, Pro802, and Tyr806 from ALIX representing as essential hydrophobic bonding. The other hydrophobic bonding was notified by Trp184 and Gln190 from CEP55 chain A, which made an Arene-H contact with Pro801 and Tyr809, respectively. Hydrogen bonding was also found between Tyr806 of ALIX-Glu192 of CEP55 and Gln799 of ALIX-Asn181 of CEP55. Despite the few H-bond profiles between CEP55 and ALIX, all of the distances were less than 2 Å, suggesting strong hydrogen bonding, which is crucial for interaction (Table 2). We then explored the possible sequence that could be considered for drug inhibitory study. Using the SiteFinder method in MOE, we obtained four considered pockets. Two sites were located on the EABR site, whereas the others were on the N-terminal site of CEP55 (Table 3, Figure 4B). Unexpectedly, Lys180, Gln183, and Leu185 were selected by SiteFinder instead of Glu192 because of the involvement of solvent contact with Gln799 (Figure 4C). The generated residues from SiteFinder were then prepared for drug inhibitor screening by molecular docking.

**Table 2:** Binding interaction between ALIX and CEP55

**ALIX**	**CEP55**		**Binding Type**	**Distance (Å)**
	**Chain A**	**Chain B**		
Gln799		Asn181	H-bond	1.76
Gln799		Asn181	H-bond	1.92
Gln799	Trp184		H-bond	1.91
Pro801	Trp184		Arene-H	
Pro801	Tyr187		Arene-H	
Pro802	Tyr187		Arene-H	
Tyr806	Tyr187		Arene-H	
Tyr806		Glu192	H-bond	1.74
Tyr809	Gln190		Arene-H	

**Table 3:** Pocket search generated from site finder program in MOE

**Site**	**Size**	**Hyd**	**Side**	**Residue**
1	68	19	55	1:(Lys180, Gln183, Trp184, Tyr187) 2:(Asn181, Leu185)
2	46	14	35	2:(Lys180, Gln183, Trp184, Trp187)
3	41	7	29	1:(His168, Glu171, Ile172, Lys175)
4	40	6	32	1:(Ile167, Met170) 2:(Ile167, Met170, Glu171)

**Table 4:** Docking score of several natural compounds toward CEP55

**Natural Compound**	**Docking Score (kcal/mol)**	**RMSD (Å)**
Epigallocatechin gallate	−10.44	0.972
Catechin	−9.69	1.169
Hesperetin	−9.19	1.161
Hesperidin	−8.57	1.579
Luteolin	−8.29	0.728
Homoharringtonine	−8.27	1.709
Resveratrol	−8.08	1.581
Curcumin	−7.84	1.198
Colchicine	−7.46	1.584
Quercetin	−7.38	1.077
Genistein	−7.36	1.826
Daidzein	−7.32	1.225
Naringenin	−7.16	1.619
Ingenol mebutate	−7.10	1.685
Vincristine	−6.81	1.778
Paclitaxel	−6.68	2.016
Vinblastine	−6.63	0.886

### Evolutionary analysis of the CEP55 gene in various vertebrates

**Figure 5:**
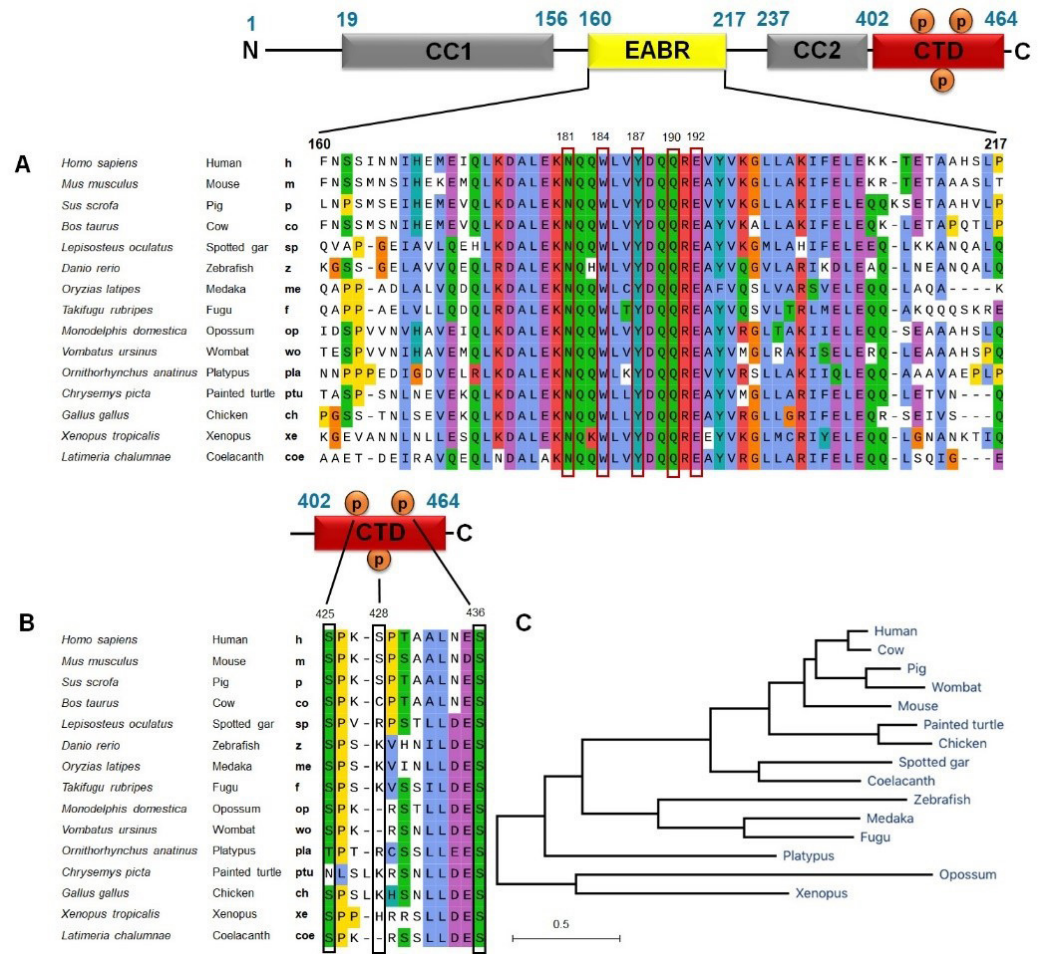
Evolutionary analysis of CEP55 genes in various species. Alignment of amino acid residues critical for ALIX binding (EABR domain) of CEP55 (A) and phosphorylation sites of C-terminal CEP55 (B). C. Phylogenetic tree of CEP55 across mammalian and non-mammalian vertebrates. CEP55 nucleotide sequences were downloaded from NCBI and Ensembl. The amino acids alignment was done using ClustalW method and the phylogenetic tree was constructed using the neighbor joining method by Mega X software.

CEP55 plays an important role, and its dysregulation promotes genomic instability contributing to tumor heterogeneity and cancer evolution. Thus, we conducted an evolutionary relationship analysis of CEP55 in various mammalian and non-mammalian vertebrate species by using MEGA software. Orthologs of human CEP55 have been identified in many but not all vertebrates, including Mammals: *Homo sapiens* (human), *Mus musculus* (mouse), *Sus scrofa* (pig), *Bos taurus* (cow), Fishes: *Lepisosteus oculatus* (spotted gar), *Danio rerio*(zebrafish), *Oryziaslatipes* (medaka), *Takifugurubripes* (fugu), *Latimeria chalumnae* (coelacanth), Marsupials: *Monodelphis domestica* (opossum), *Vombatus ursinus* (wombat), Reptiles: *Chrysemyspicta* (painted turtle), Birds: *Gallus* (chicken), Amphibians: *Ornithorhynchus anatinus* (platypus), and *Xenopus tropicalis*(xenopus). Analysis of amino acid sequence alignment showed that CEP55 has a degree of similarity. Most amino acid residues essential for ALIX (EABR domain), a portion responsible for interacting with either ALIX or TSG101 by CEP55, were conserved among vertebrate species (Figure 5A). Molecular docking results showed that the amino acid residues are responsible for hydrophobic and hydrogen binding include Asn181 (Asparagine/N), Trp184 (Tryptophan/W), Tyr187 (Tyrosine/Y), Gln190 (Glutamine/Q), and Glu192 (Glutamic acid/E) (Table 2).

As described above, the phosphorylation sites of N-terminal CEP55 are crucial for its localization in the midbody. Figure 5B shows that Ser436, target of PLK1 phosphorylation, is highly conserved across all species. The two other phosphorylation sites required for the second phosphorylation, Ser425, is also conserved among species, except in painted turtles (*C. picta*). We then constructed a phylogenetic tree by using the nucleotide sequences of coding regions in these species. The phylogenetic tree analysis of CEP55 demonstrated clear differences in the evolutionary rates among all vertebrates, indicated by the branch length of the rooted tree (Figure 5C). Human CEP55 is phylogenetically closer to other mammals and wombat as clustered in one branch. In the fish family, zebrafish–medaka–fugu and spotted gar–coelacanth are in one group. Evolutionary analysis of CEP55 suggests that despite the diversification across species, the important amino acid residues of CEP55 are highly conserved and critical for CEP55 function.

### Screening of CEP55 inhibitor by molecular docking

**Figure 6:**
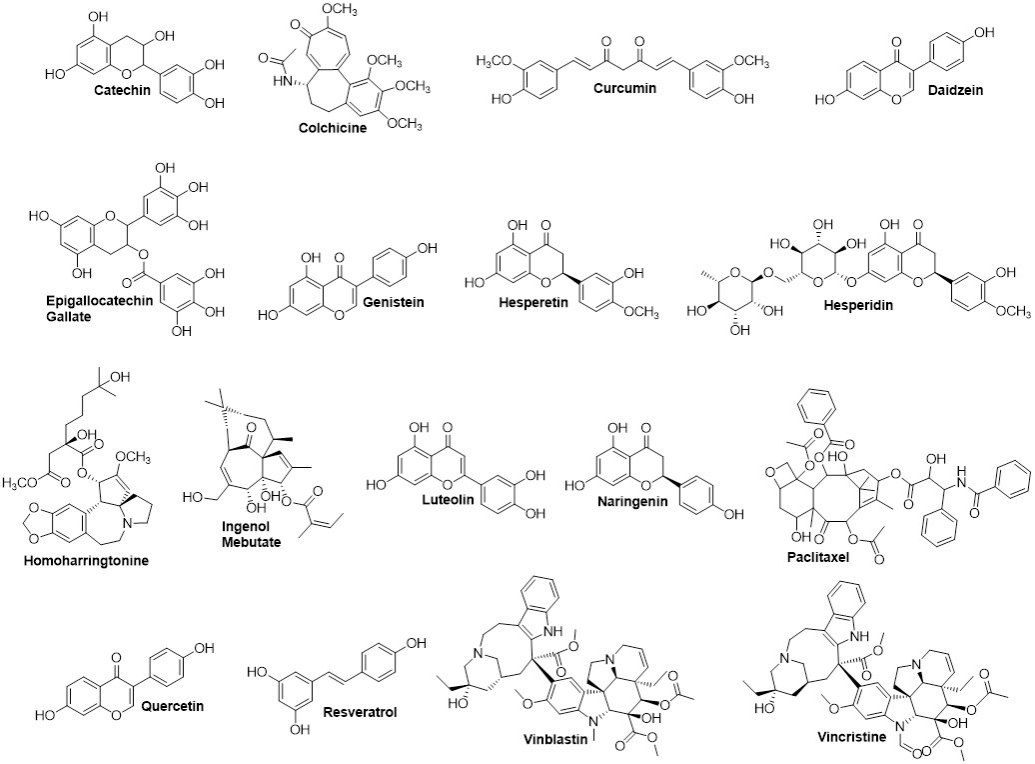
Chemical structure of natural compound screened for CEP55 inhibitor using Molecular Docking analysis. Those 17 natural compounds: Epigallocatechin gallate, catechin, hesperetin, hesperidin, luteolin, homoharringtonine, resveratrol, curcumin, colchicine, quercetin, genistein, daidzein, naringenin, ingenol mebutate, vincristine, paclitaxel, vinblastine have been entering/passing clinical trials as anticancer agents.

**Figure 7:**
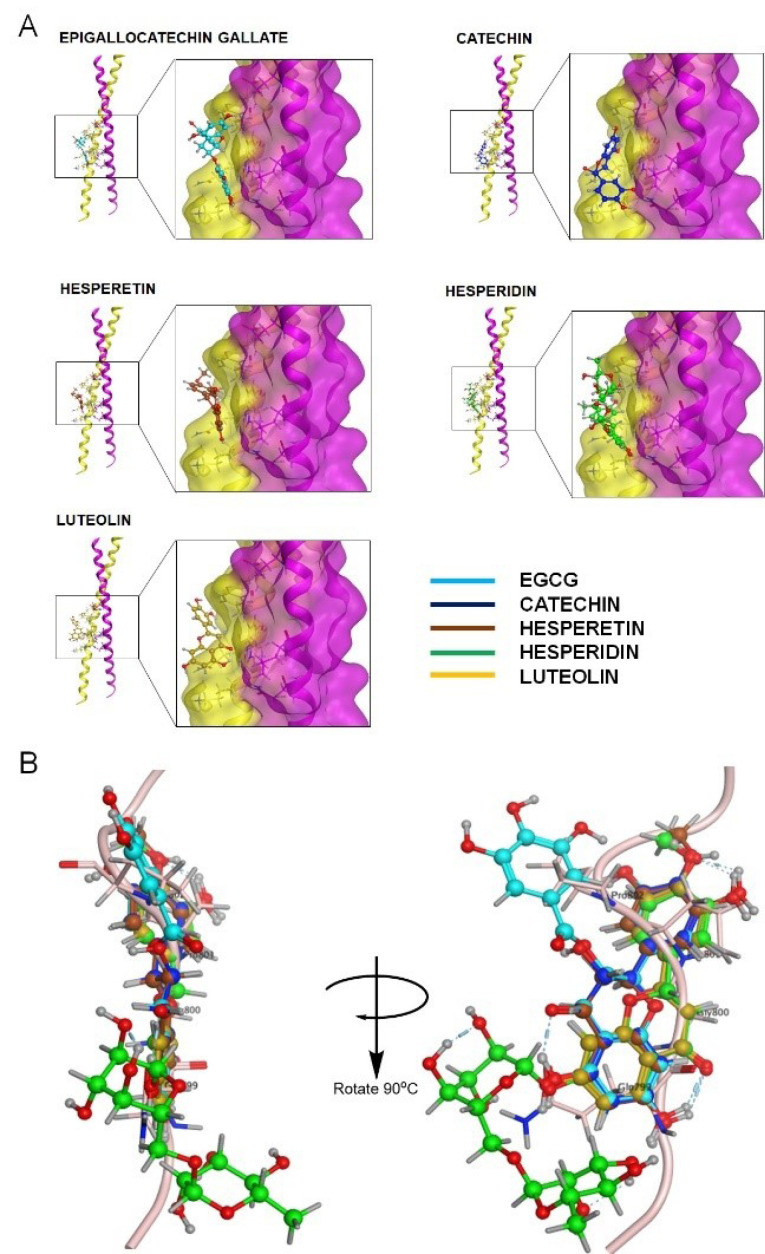
Visualization of interaction of top five natural compounds on CEP55. A. Profile of binding interaction between the representative of compounds with CEP55 on ALIX-CEP55 binding site. B. Structural alignment of the top five compounds with ALIX protein Homodimer or coiled-coil of CEP55 was shown in yellow and magenta protein ribbons, while the light pink ribbon represented the ALIX protein. Each compound was visualized in blue-colored ball and stick chain.

Our screening on defining the drug-able site for inhibitory study using Site Finder produced four possible sites. Each site was used for molecular docking to screen the favorable inhibitors from 17 natural compounds entering or passing clinical trials as anticancer agents (Figure 6). Our screening revealed the top five compounds represented by flavonoids, particularly flavanol, flavanone, and flavanone; EGCG performed the lowest docking score (-10.44 kcal/mol), followed by catechin, hesperetin, hesperidin, and luteolin, indicating the high affinity (Table 4). Furthermore, these compounds bound to the ALIX–CEP55 binding site and disrupted the protein–protein interaction (Figure 7A). Other flavonoid compounds classified as isoflavones, such as genistein, daidzein, and quercetin, showed low affinity as indicated by high docking scores. After structural alignment, the top five compounds mimicked the structure of the GPP sequence of ALIX (Figure 7B). Taken together, the results revealed that flavanol class compounds EGCG and catechin are the most promising candidates and important structures to be considered for further derivative screening.

## Discussion

Mammalian cells can be accurately divided into two daughter cells after passing proper cytokinesis. Cytokinesis has been extensively explored, and several lines of evidence from previous research suggest that CEP55 is involved in the regulation of cytokinesis. To our knowledge, no inhibitors of CEP55 have been developed yet. Meanwhile, several selective inhibitors of PLK1, another protein involved in cytokinesis, have been generated and entered clinical studies. A phase III clinical trial for volasertib, an ATP-competitive PLK1 inhibitor, has shown considerable promise for combating cancer cells. In the present study, we found CEP55 upregulation in a large collection of cancer types compared with their normal counterparts, highlighting their association with poor prognosis in cancer patients. The bioinformatics databases from Oncomine and TCGA summarized that CEP55 amplification is more frequently found in a wide range of tumor types than deep deletion events. These findings provide improved knowledge about CEP55 mechanism and suggest that inhibiting CEP55 localization and/or function is a potential strategy for cancer treatment.

The present study showed strong evidence that the functional domains of CEP55 are highly conserved in several mammalian and non-mammalian species. The residues of asparagine181, tryptophan184, tyrosine187, glutamine190, and glutamic acid192, which exhibit membrane scission activity, are important for binding with the ESCRT complex. Conversely, the highly conserved serine residues do not contribute in CEP55 function but in the recruitment to the midbody, including serine425 and serine436. Fabbro et al reported that mutation of Cep55 phosphorylation sites at serine425 and serine428 prevents the CEP55 dissociation from the centrosome at the G2/M boundary.^19^ Moreover, ser436 is PLK1-dependent phosphorylation, a very well-known kinase protein in mitotic phase.^30^ The impairment of PLK1 phosphorylation leads to abnormal abscission defects and disrupts the recruitment of ESCRT machinery to the midbody.^31,32^ Those important residues of CEP55 might promise positive clues for designing anticancer drugs. The combination of CDK1/ERK2/PLK1 and CEP55 inhibitors is a rational strategy to impair cell cancer division and merits further investigation.

**Figure 8:**
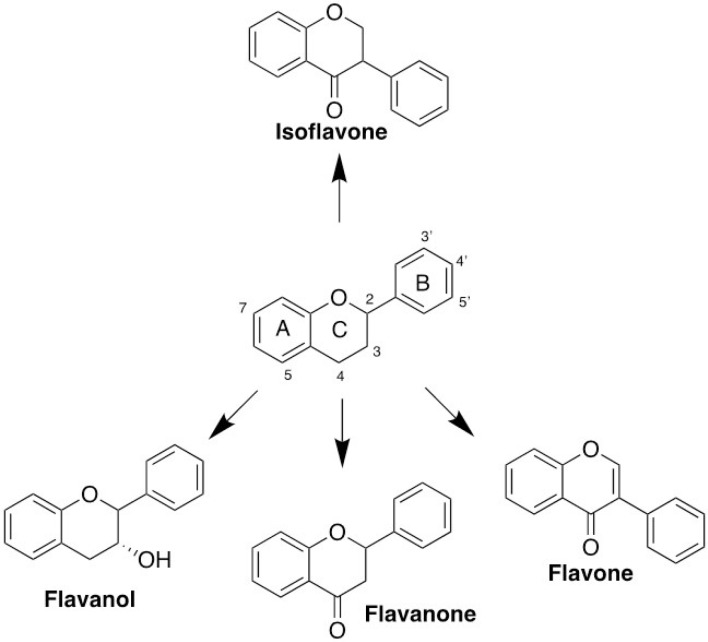
General classification of flavonoid compounds.

Over the past two decades, an extensive and intensive research for drugs targeting cell cycle proteins have led to the identification of many candidates of small-molecule inhibitors. Our molecular docking screening in this study found that several flavonoid classes, such as flavanol, flavanone, and flavone, are more promising candidates than isoflavones or other natural compound classes for disrupting ALIX–CEP55 interaction (Figure 8). Those three flavonoid classes recognized by the substitution of chain B on C2 position are more favorable to mimic ALIX structure than isoflavones, which place chain B as the substituent on C3 position. Flavanol compounds EGCG and catechin possessed superior binding affinity among others, indicating the important role of gallic ester or hydroxyl group substitution on C3 position as previously reported.^33^ Gallic ester or hydroxyl group substitution not only provides H-bond formation through its hydroxyl group but also contributes on hydrophobic binding with several aromatic amino acid residues.^34,35^ The presence of ketone group on C4 position of chain C decreases the binding affinity as represented by flavanone and flavone. In addition, the binding affinity of flavanone compounds hesperidin and hesperetin is higher than that of flavone compound luteolin because of the presence of methoxy group on the C4’ position. The methoxy group of hesperidin or hesperitin through its methoxy contributes to its flexibility to bind in the protein binding site.^36^ Overall, this study reveals the important structure of the CEP55 binding protein and serves as a reference for the design of small-molecule CEP55 inhibitors. The next research should be directed toward designing a specific and potent CEP55 inhibitor followed by *in vitro* and *in vivo* studies.

## Conclusion

Here, we propose CEP55 as the considerable target for anticancer drugs and cancer biomarkers because of the massive upregulation and unfavorable clinical features. We also suggest the use of EGCG or catechin as an inhibitor of CEP55 and an important structure for further drug design.

## Ethical Issues

Not applicable.

## Conflict of Interest

The authors declare that there is no conflict of interest.

## Acknowledgments

We thank Prof. Dr. Edy Meiyanto, M.Si., Apt. (Universitas Gadjah Mada) for the valuable discussion and comments in the preparation of this manuscript. We also thank Enago (https://www.enago.com) for English-language editing.

## References

[1] Jackson JR, Patrick DR, Dar MM, Huang PS (2007). Targeted anti-mitotic therapies: can we improve on tubulin agents?. Nat Rev Cancer.

[2] Dumontet C, Jordan MA (2010). Microtubule-binding agents: a dynamic field of cancer therapeutics. Nat Rev Drug Discov.

[3] Kastan MB, Bartek J (2004). Cell-cycle checkpoints and cancer. Nature.

[4] Mierzwa B, Gerlich DW (2014). Cytokinetic abscission: molecular mechanisms and temporal control. Dev Cell.

[5] Jeffery J, Sinha D, Srihari S, Kalimutho M, Khanna KK (2016). Beyond cytokinesis: the emerging roles of CEP55 in tumorigenesis. Oncogene.

[6] Kalimutho M, Sinha D, Jeffery J, Nones K, Srihari S, Fernando WC (2018). CEP55 is a determinant of cell fate during perturbed mitosis in breast cancer. EMBO Mol Med.

[7] Lee HH, Elia N, Ghirlando R, Lippincott-Schwartz J, Hurley JH (2008). Midbody targeting of the ESCRT machinery by a noncanonical coiled coil in CEP55. Science.

[8] Morita E, Sandrin V, Chung HY, Morham SG, Gygi SP, Rodesch CK (2007). Human ESCRT and ALIX proteins interact with proteins of the midbody and function in cytokinesis. EMBO J.

[9] Li F, Jin D, Tang C, Gao D (2018). CEP55 promotes cell proliferation and inhibits apoptosis via the PI3K/Akt/p21 signaling pathway in human glioma U251 cells. Oncol Lett.

[10] Chen CH, Lu PJ, Chen YC, Fu SL, Wu KJ, Tsou AP (2007). FLJ10540-elicited cell transformation is through the activation of PI3-kinase/AKT pathway. Oncogene.

[11] Pan TL, Hsu SY, Wang PW, Cheng YT, Chang YC, Saha S (2015). FLJ25439, a novel cytokinesis-associated protein, induces tetraploidization and maintains chromosomal stability via enhancing expression of endoplasmic reticulum stress chaperones. Cell Cycle.

[12] Chou TC, Zhang X, Zhong ZY, Li Y, Feng L, Eng S (2008). Therapeutic effect against human xenograft tumors in nude mice by the third generation microtubule stabilizing epothilones. Proc Natl Acad Sci U S A.

[13] Cragg GM, Katz F, Newman DJ, Rosenthal J (2012). The impact of the United Nations Convention on Biological Diversity on natural products research. Nat Prod Rep.

[14] Butler MS (2004). The role of natural product chemistry in drug discovery. J Nat Prod.

[15] Vijayakumar S, Manogar P, Prabhu S, Sanjeevkumar Singh RA (2018). Novel ligand-based docking; molecular dynamic simulations; and absorption, distribution, metabolism, and excretion approach to analyzing potential acetylcholinesterase inhibitors for Alzheimer’s disease. J Pharm Anal.

[16] Hermawan A, Putri H, Utomo RY (2020). Comprehensive bioinformatics study reveals targets and molecular mechanism of hesperetin in overcoming breast cancer chemoresistance. Mol Divers.

[17] Kusumastuti R, Utomo RY, Khumaira A, Putri H, Jenie RI, Meiyanto E (2019). Pentagamaboronon-0 increased cytotoxicity of and inhibited metastasis induction by doxorubicin in breast cancer cells. J Appl Pharm Sci.

[18] Min K, Lee HH (2016). Molecular aspects of CEP55 during cytokinesis and tumorigenesis. Biodesign.

[19] Fabbro M, Zhou BB, Takahashi M, Sarcevic B, Lal P, Graham ME (2005). Cdk1/Erk2- and Plk1-dependent phosphorylation of a centrosome protein, Cep55, is required for its recruitment to midbody and cytokinesis. Dev Cell.

[20] Said Halidi KN, Fontan E, Boucharlat A, Davignon L, Charpentier M, Boullé M (2019). Two NEMO-like ubiquitin-binding domains in CEP55 differently regulate cytokinesis. iScience.

[21] van der Horst A, Simmons J, Khanna KK (2009). Cep55 stabilization is required for normal execution of cytokinesis. Cell Cycle.

[22] Wang Y, Jin T, Dai X, Xu J (2016). Lentivirus-mediated knockdown of CEP55 suppresses cell proliferation of breast cancer cells. Biosci Trends.

[23] Sinha D, Nag P, Nanayakkara D, Duijf PHG, Burgess A, Raninga P (2020). Cep55 overexpression promotes genomic instability and tumorigenesis in mice. Commun Biol.

[24] Qi J, Liu G, Wang F (2018). High levels of centrosomal protein 55 expression is associated with poor clinical prognosis in patients with cervical cancer. Oncol Lett.

[25] Xiao H, Xu D, Chen P, Zeng G, Wang X, Zhang X (2018). Identification of five genes as a potential biomarker for predicting progress and prognosis in adrenocortical carcinoma. J Cancer.

[26] Singh PK, Srivastava AK, Rath SK, Dalela D, Goel MM, Bhatt ML (2015). Expression and clinical significance of Centrosomal protein 55 (CEP55) in human urinary bladder transitional cell carcinoma. Immunobiology.

[27] Waseem A, Ali M, Odell EW, Fortune F, Teh MT (2010). Downstream targets of FOXM1: CEP55 and HELLS are cancer progression markers of head and neck squamous cell carcinoma. Oral Oncol.

[28] Wu S, Wu D, Pan Y, Liu H, Shao Z, Wang M (2019). Correlation between EZH2 and CEP55 and lung adenocarcinoma prognosis. Pathol Res Pract.

[29] Peng T, Zhou W, Guo F, Wu HS, Wang CY, Wang L (2017). Centrosomal protein 55 activates NF-κB signalling and promotes pancreatic cancer cells aggressiveness. Sci Rep.

[30] Bastos RN, Barr FA (2010). Plk1 negatively regulates Cep55 recruitment to the midbody to ensure orderly abscission. J Cell Biol.

[31] Chang YC, Wu CH, Yen TC, Ouyang P (2012). Centrosomal protein 55 (Cep55) stability is negatively regulated by p53 protein through Polo-like kinase 1 (Plk1). J Biol Chem.

[32] Stoten CL, Carlton JG (2018). ESCRT-dependent control of membrane remodelling during cell division. Semin Cell Dev Biol.

[33] Fang MZ, Wang Y, Ai N, Hou Z, Sun Y, Lu H (2003). Tea polyphenol (-)-epigallocatechin-3-gallate inhibits DNA methyltransferase and reactivates methylation-silenced genes in cancer cell lines. Cancer Res.

[34] Wang Y, Ren X, Deng C, Yang L, Yan E, Guo T (2013). Mechanism of the inhibition of the STAT3 signaling pathway by EGCG. Oncol Rep.

[35] Chen H, Yao K, Chang X, Shim JH, Kim HG, Malakhova M (2015). Computational and biochemical discovery of RSK2 as a novel target for epigallocatechin gallate (EGCG). PLoS One.

[36] Ding F, Peng W (2015). Biological activity of natural flavonoids as impacted by protein flexibility: an example of flavanones. Mol Biosyst.

